# Ultrasound had high accuracy in measuring hip joint capsule thickness

**DOI:** 10.1186/s12891-024-07228-0

**Published:** 2024-01-29

**Authors:** Guanying Gao, Huaan Fang, Kaicheng Zhou, Zizhi Mo, Jiayang Liu, Lingyu Meng, Jianquan Wang, Yan Xu

**Affiliations:** Institute of Sports Medicine, Beijing Key Laboratory of Sports Injuries, Peking University Third Hospital, 49 North Garden Road, Haidian District, Beijing, 100191 China

**Keywords:** Ultrasound, Hip, Capsule, Magnetic resonance imaging

## Abstract

**Background:**

The hip joint capsule is an essential component of hip joint function and stability, and its thickness is closely associated with certain medical conditions, surgical outcomes, and rehabilitation treatments. Currently, in clinical practice, hip joint capsule thickness is predominantly measured using magnetic resonance imaging (MRI), with limited utilization of ultrasound examinations for this purpose.

**Methods:**

We retrospectively evaluated patients who visited our Sports Medicine Department between February 2017 and March 2023 and underwent both hip joint MRI and ultrasound imaging on the same side. All patients had undergone preoperative hip joint MRI and ultrasound examinations, with the time gap between the two examinations not exceeding three months. Measurements of hip joint capsule thickness were taken on both MRI and ultrasound images for the same patients to analyze their consistency. Additionally, we measured the alpha angle, lateral center-edge angle, acetabular anteversion angle, and femoral anteversion angle of the patients’ hip joints and analyzed their correlation with hip joint capsule thickness measure by ultrasound.

**Results:**

A total of 307 patients were included in this study, with hip joint capsule thickness measured by MRI and ultrasound being 5.0 ± 1.2 mm and 5.0 ± 1.5 mm, respectively. The Bland-Altman analysis demonstrates good agreement or consistency. The paired t-test resulted in a p-value of 0.708, indicating no significant statistical difference between the two methods. The correlation analysis between acetabular anteversion angle and ultrasound-measured capsule thickness yielded a p-value of 0.043, indicating acetabular anteversion angle and capsular thickness may have negative correlation.

**Conclusions:**

The measurements of joint capsule thickness obtained through ultrasound and MRI showed good consistency, suggesting that ultrasound can be used in clinical practice as a replacement for MRI in measuring hip joint capsule thickness. There was a significant correlation between acetabular anteversion angle and hip joint capsule thickness, indicating potential for further research in this area.

## Introduction

The hip joint capsule is an essential component of hip joint function and stability. Its role becomes particularly pronounced when potential bone, cartilage, or soft tissue damage leads to diminished hip joint stability [1, 2]. Biomechanical evidence suggests that the hip joint capsule plays a crucial role in hip joint stability. The iliofemoral ligament, pubofemoral ligament, and ischiofemoral ligament are integral components of the hip joint capsule, providing constraints and stability to various aspects of hip joint motion, including internal and external rotation, extension, and more.

The joint capsule also plays a crucial role in various biological functions, and its morphology and dimensions to some extent indicate the severity of a patient’s condition [3–5]. Furthermore, arthroscopic surgery is a commonly employed minimally invasive technique in clinical practice for treating various conditions. It is important to note that arthroscopic surgery may cause some degree of damage to the joint capsule [6, 7]. Therefore, selecting an appropriate method to assess the morphology of the joint capsule is an important factor in clinical planning, evaluating treatment outcomes, and selecting suitable rehabilitation strategies.

In clinical practice, magnetic resonance imaging (MRI) is commonly utilized to assess the morphology of joint capsules. However, MRI has its limitations, including time-consuming procedures, causing claustrophobia and high costs, which, in certain situations, can impede a prompt and cost-effective evaluation of the joint capsule’s status. Ultrasound offers several advantages over MRI, including its cost-effectiveness, convenience, speed, flexibility, and comfort. In clinical practice, ultrasound is often used as an adjunctive tool for procedures such as surgeries and hip joint injections [8–10]. The current researches regarding the assessment of the hip joint capsule using ultrasound remain limited, and the feasibility of utilizing ultrasound to assess the morphology of the hip joint capsule has not been validated.

The purpose of this study was to evaluate the feasibility of using ultrasound examinations as a substitute for MRI in the clinical assessment of hip joint capsule thickness. We hypothesized that ultrasound and MRI had good consistency and ultrasound can be used in clinical practice as a replacement for MRI in measuring hip joint capsule thickness.

## Method

### Patients

We conducted a retrospective assessment of patients who attended our Sports Medicine Clinic between February 2017 and March 2023. Inclusion criteria were as follows: (1) availability of preoperative ultrasound and MRI results for the same-side hip joint; (2) a time difference of no more than 3 months between the ultrasound and MRI examinations. Patients with previous hip surgery were excluded from this study. Informed consent was obtained from all enrolled patients. This study has received approval from the Ethics Committee of our hospital.

### Magnetic resonance imaging

Magnetic resonance imaging (MRI) analysis was performed by a radiologist specializing in musculoskeletal diseases with over ten years of experience in musculoskeletal radiology. The radiologist conducting the MRI analysis was blinded to the ultrasound results as described previously [11]. In brief, patients were in a supine position, and conventional MRI of the affected hip joint was obtained. Hip joint MRI examinations were conducted using a 3.0 T MRI scanner (Magnetom Trio with TIM system, Siemens Healthcare) and a dedicated flexible surface coil positioned around the affected hip joint. Fat-suppressed turbo spin-echo intermediate sequences and T2-weighted sequences were acquired separately in axial, oblique transverse, and oblique coronal planes. The oblique transverse imaging plane was oriented parallel to the axis of the femoral neck, while the oblique coronal plane imaging was oriented perpendicular to the line through the anterior and posterior edges of the acetabulum on the axial images. Conventional Turbo spin-echo T1-weighted sequences were routinely acquired in the oblique coronal plane. The total imaging time for one hip joint MRI examination was 30 to 35 min.

### Ultrasound examination

The ultrasound examination was performed by a radiologist with extensive experience, who was also blinded to the MRI results. As described previously [12], Patients were in a supine position with the hip slightly externally rotated. Longitudinal images were obtained in the transverse oblique plane, parallel to the axis of the femoral neck, to identify the acetabular roof, joint capsule, acetabular labrum, and femoral head and neck. The transducer was then moved to the medial and lateral aspects of the hip joint to assess the anterior quadrant of the acetabular labrum. The total time taken for one hip joint ultrasound examination was 5–10 min.

### Measurement

#### Capsular thickness on MRI

**Fig. 1:**
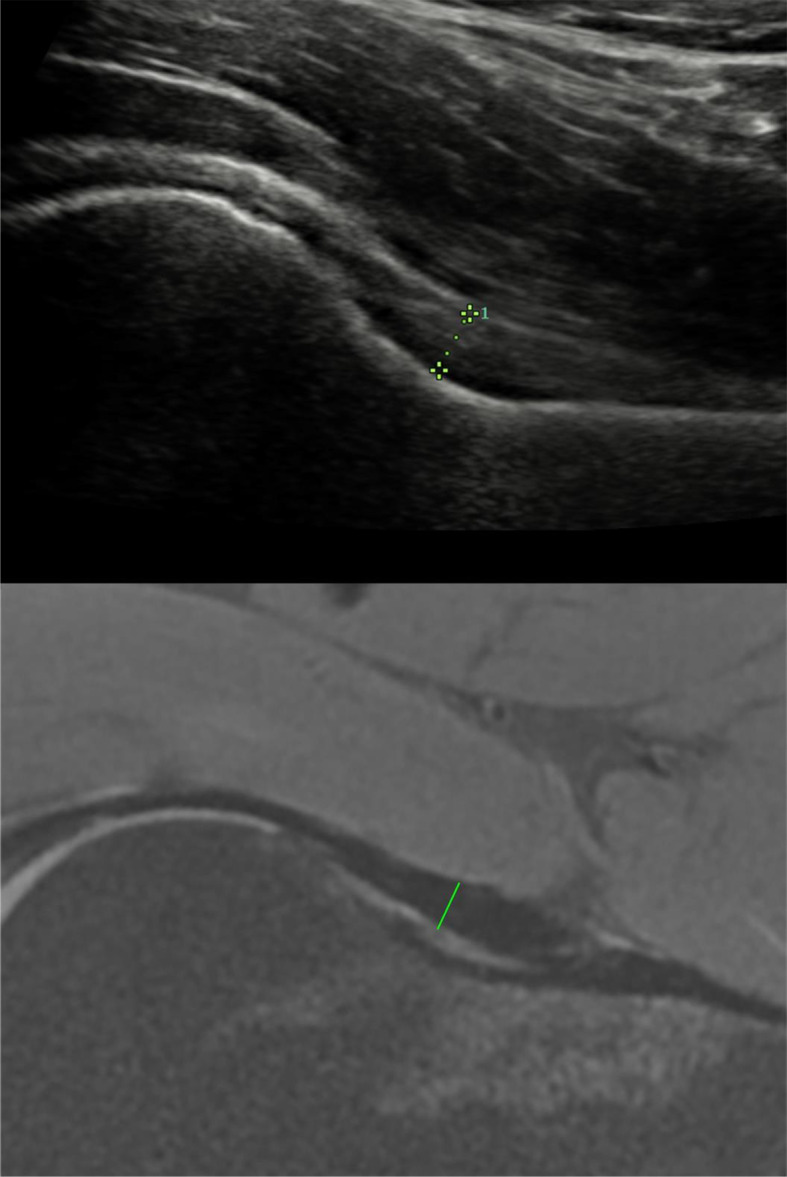
Left hip capsule thickness measurement of same patient on MRI and ultrasound

Capsular thickness in this study means tissue dimensions of capsular ligaments. The measurement of the thickness of the joint capsule by MRI was conducted by two surgeons, who were unaware of the ultrasound measurements of the hip joint capsule during the measurements to avoid potential bias. As described by Strickland et al. [13], hip joint capsule thickness was measured on coronal plane at the level of the femoral head–neck junction (Fig. 1). The capsule thickness was calculated by measuring the low-signal intensity substance between the joint side and the muscle side.

#### Capsular thickness on ultrasound

The measurement of hip joint capsule thickness by ultrasound was conducted by two radiologists, blinded to the results of MRI, who specializes in musculoskeletal disorders with more than 10 years of experience. The same plane as the MRI imaging was selected (Fig. 1), and the thickness of the joint capsule at the junction of the femoral head and neck was measured. The thickness of the capsule was calculated by measuring the hyperechoic substance between the joint side and the muscle side.

#### Alpha angle, lateral center–edge angle (LCEA), femoral neck anteversion and central acetabular version

Alpha angle, lateral center-edge angle (LCEA), femoral neck anteversion and central acetabular version of the patients were also measured in this assessment, following the methods described in previous studies [14–17]. The alpha angle was measured on plain radiographs in the Dunn view position of the affected hip. The LCEA was measured on the anteroposterior (AP) pelvis radiograph. Femoral anteversion and central acetabular version were measured on hip and knee CT.

### Statistics

We conducted a Bland-Altman analysis [18]. to assess the agreement between joint capsule thickness measurements obtained through ultrasound and MRI. The average difference is determined by calculating the mean of the measurements obtained from MRI and ultrasound. The Bland-Altman analysis yielded the following descriptive statistics: mean difference, the standard deviation of the differences (SD), and the 95% upper and lower limits of agreement. We generated a scatter plot with “Mean” on the X-axis and “Difference” on the Y-axis. The plot included reference lines on the Y-axis as follows: the mean of the differences (mean), the upper limit of agreement (mean + 1.96 * SD), and the lower limit of agreement (mean − 1.96 * SD). The Bland-Altman plot visually represents the relationship between the differences and the mean values.

A two-tailed paired t-test was employed to assess the concordance between joint capsule thickness measurements obtained through ultrasound and those obtained through MRI. Additionally, a correlation analysis was conducted to validate the consistency of capsule thickness measurements between MRI and ultrasound. In addition, we also conducted a correlation analysis between variables such as femoral anteversion and central acetabular version, and hip joint capsule thickness to observe whether there is any relationship between them. Interrater reliability was evaluated using a two-way, mixed, absolute-agreement, single-measures intraclass correlation coefficient (ICC). A p-value of < 0.05 was considered statistically significant. All statistical analyses were performed using SPSS Statistics version 22 (IBM).

## Results

### Patient demographics

This study initially included 429 patients. After excluding patients who did not have preoperative hip joint ultrasound and MRI images on the same side or whose preoperative hip joint ultrasound and MRI images differed by more than 3 months, a total of 307 patients were ultimately included (mean age 38.6 years, age range: 13–67 years; 125 males and 182 females). The demographic characteristics of the included patients, including age, sex, side, BMI, alpha angle, LCEA, and preoperative hip joint capsule thickness measured by ultrasound and MRI, are presented in Table 1. The ICC for capsular thickness by ultrasound and MRI between 2 evaluators was 0.91, and 0.94, respectively.

**Table 1:** Patient demographics

Parameter	Data
Age, y, mean (range)	38.6(13–67)
*Sex*	
Male	125(19–67)
Female	182(13–65)
*Side*	
Left	151(male: *n* = 53; female: *n* = 98)
Right	157(male: *n* = 72; female: *n* = 85)
BMI, kg/m², mean(range)	23.0(16.7–33.2)
Alpha angle, mean ± SD	57.9 ± 9.7
LCEA, mean ± SD Femoral neck anteversion, mean ± SD Acetabular anteversion angle, mean ± SD	33.5 ± 6.7 19.6 ± 8.3 18.4 ± 6.3
Preoperative capsular thickness, mm, mean ± SD	
MRI	5.0 ± 1.2
Ultrasound	5.0 ± 1.5

Unless otherwise specified, data are numbers of patients, with percentages in parentheses; SD, Standard deviation

### Comparison of hip joint capsule thickness measurements obtained through ultrasound and MRI

**Fig. 2:**
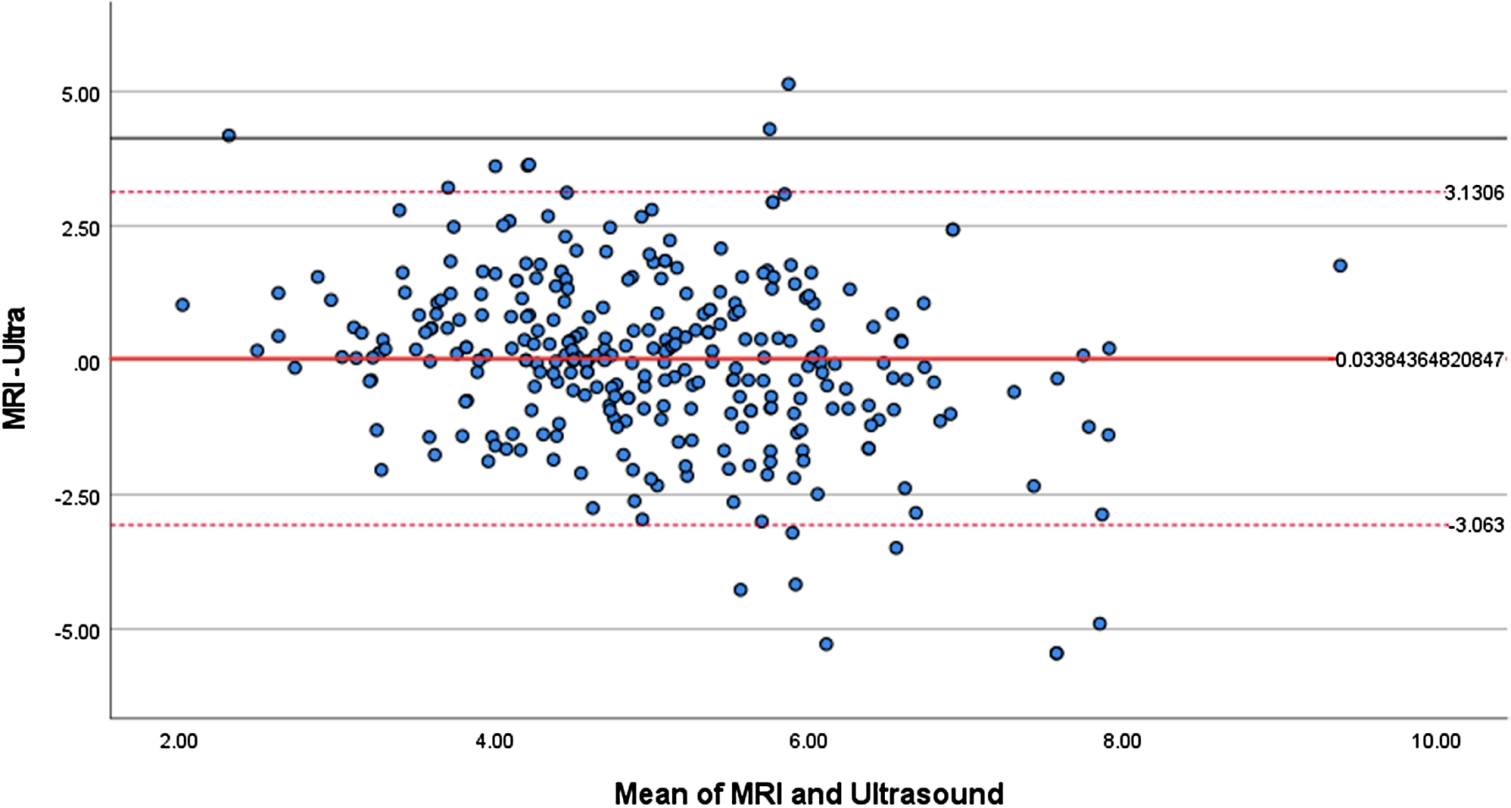
Bland-Altman plots show differences between two measurement methods. Dashed lines show 95% limits of agreement (± 1.96 SD); solid line, mean of differences

A Bland-Altman analysis was performed on hip joint thickness measurements from MRI and ultrasound for 307 patients (Fig. 2). The Bland-Altman plot illustrates the concordance between ultrasound and MRI measurements of joint capsule thickness. The X-axis represents the average values of the two measurement methods, while the Y-axis represents the difference values. The mean difference (MRI - Ultra) was found to be 0.0338, with a standard deviation (SD) of 1.58. It can be observed that the majority of data points fall within the limits of agreement, with a percentage difference of 297/307, which is 95.8%. This indicates that in most cases, the two measurement methods exhibit agreement or consistency.

Correlation and a two-tailed paired t-test were conducted on the hip joint capsule thickness measured by MRI and ultrasound for the 307 patients. The p-value for the paired sample correlation was less than 0.001, indicating a significant correlation between the two variables. The p-value for the paired sample t-test was 0.708, suggesting that there is no significant difference in the means of the two variables in a statistical sense.

### The correlation between alpha angle, LCEA, femoral neck anteversion, acetabular anteversion angle with hip joint capsule thickness

Correlation analyses were conducted between alpha angle and hip joint capsule thickness, LCEA and hip joint capsule thickness, femoral neck anteversion and hip joint capsule thickness, and acetabular version and hip joint capsule thickness. Mean hip joint capsule thickness data obtained from ultrasound were used for the analysis. The p-values obtained from the correlation analysis were 0.139, 0.731, 0.887, and 0.043, respectively. The analysis indicates a significant correlation between acetabular version and hip joint capsule thickness, while the other three sets of data showed no significant correlation with hip joint capsule thickness. Acetabular anteversion angle and capsular thickness may have negative correlation.

## Discussion

Our study conducted a consistency analysis of hip joint capsule thickness measurements obtained using ultrasound and MRI. The results demonstrated good consistency between the two methods, suggesting that ultrasound can be used as a clinical alternative to MRI for measuring hip joint capsule thickness. We also examined the correlations between alpha angle, LCEA, femoral neck anteversion, central acetabular version with joint capsule thickness. Data analysis revealed a significant correlation between acetabular anteversion and MRI joint capsule thickness. This correlation could potentially assist in our further exploration of hip joint stability and provide recommendations for surgical planning and treatment outcomes. However, no significant correlation was found between femoral neck anteversion and joint capsule thickness. Future research may involve further data selection and an increased sample size for femoral neck anteversion to conduct more in-depth investigations.

The hip joint capsule is an essential component of the hip joint, and anatomical studies on cadavers have demonstrated its significant role in both the function and stability of the hip joint [19, 20]. Within the hip joint capsule, ligaments such as the iliofemoral ligament, ischiofemoral ligament, and pubofemoral ligament play dominant roles in ensuring the functional mobility and stability of the hip joint. The circular bands within the hip joint capsule also resist joint distraction, thus contributing to joint stability [21]. Therefore, the assessment of hip joint capsule dimensions plays a crucial role in diagnosing hip joint diseases, evaluating treatment outcomes, and maintaining joint functionality.

In recent years, there has been a growing recognition of the importance of managing the hip joint capsule appropriately during surgical interventions for hip joint diseases [22–24]. Increasing research efforts have been directed toward aspects of hip joint capsule management during hip surgeries, including the site of capsular incision, whether to close the capsule postoperatively, and other factors. The aim is to reduce complications such as capsular laxity or tears resulting from surgery, thereby preserving postoperative joint stability.

Currently, the commonly used method for assessing joint capsule morphology in clinical practice is MRI [25]. Previous studies have indicated that the intraclass correlation coefficient (ICC) for measurements obtained through MRI approaches approximately 0.948 [3]. Other studies utilizing MRI for measuring capsule thickness have also demonstrated good reliability. Therefore, MRI measurements of capsule thickness can reliably estimate the actual joint capsule thickness, and MRI is frequently used in clinical settings for this purpose. However, MRI does have its limitations. MRI is relatively expensive, which may result in high medical costs. MRI examinations are more time-consuming, often taking 30–35 min, during which patients need to remain still in a confined space. This can cause discomfort for patients, and individuals with claustrophobia or severe anxiety disorders may not be suitable candidates for MRI examinations. Additionally, patients with metal implants are not suitable candidates for MRI due to the strong magnetic field used in the imaging process.

In clinical practice, ultrasound is often used as an adjunctive tool for procedures such as surgeries and hip joint injections [8–10]. Ultrasound is also employed to identify various hip joint conditions, including developmental dysplasia of the hip, hip joint effusion, bursitis, and slipped capital femoral epiphysis, among others, playing a significant role in clinical diagnosis. Ultrasound offers advantages such as convenience, cost-effectiveness, high flexibility, and rapid diagnosis. However, there is relatively limited research on using ultrasound for the assessment of the hip joint capsule compared to other imaging modalities like MRI. One study examined the hip joint capsules of six pediatric cadavers using ultrasound and histological examination, assessing the anatomical and histological differences in the hip joint capsules of children with transient synovitis and those without synovitis [26]. Apart from this study, there is limited literature available regarding the use of ultrasound for hip joint capsule assessment.

In many situations, ultrasound is more suitable than MRI. It can be particularly valuable when patients experience severe claustrophobia, anxiety, or have metal implants that make them unsuitable for MRI examinations. Additionally, in emergency cases where a rapid diagnosis is needed, or when patients have financial constraints that make MRI exams difficult to afford, ultrasound can provide a clinical alternative. It helps improve the overall medical experience and serves as one of the diagnostic tools in such scenarios.

Our study also has some limitations. Different MRI or ultrasound devices can indeed have an impact on measurement results. The main concern lies in the resolution, as it can affect the accuracy of the measurements. In our study, we did not utilize multiple devices for examination. In future research, it would be beneficial to explore and analyze the variations that may arise from using different devices. Besides, we focused on using high-resolution ultrasound devices to ensure clear distinction between muscles and joint capsules. We did not measure the results at low resolutions as it could potentially hinder the accuracy of the measurements. The clinical significance represented by the consistency between MRI and ultrasound measurements of hip joint capsule thickness requires further exploration, with ongoing efforts to improve and optimize clinical treatments.

## Conclusion

This study has demonstrated good consistency between ultrasound and MRI measurements of hip joint capsule thickness, suggesting that ultrasound can be used clinically as a substitute for MRI in assessing hip joint capsule thickness. Additionally, we have identified a significant correlation between acetabular anteversion and hip joint capsule thickness, which may guide our further research efforts.

## Data Availability

All relevant data supporting the conclusions are included within the article and tables. The datasets used and/or analysed during the current study available from the corresponding author on reasonable request.
